# Minimally invasive *in vivo* endoscopic monitoring of dextran sulfate sodium-induced murine colitis

**DOI:** 10.1016/j.mex.2022.101744

**Published:** 2022-05-27

**Authors:** Elizabeth A. Bullard, Ariel I. Mundo, Shelby N. Bess, Kathryn Miller Priest, Timothy J. Muldoon

**Affiliations:** Department of Biomedical Engineering, University of Arkansas, Fayetteville, AR, United States

**Keywords:** Dextran sulfate sodium, Ulcerative colitis, Endoscopy

## Abstract

Ulcerative colitis (UC) is a gastrointestinal, autoimmune disease that causes ulceration and inflammation of the colon with an incidence of 10 out of every 100,000 people in North America and Western Europe. Though the specific cause is unknown, several studies have demonstrated that inflammatory cells as well as environmental variables, genetics, and lifestyle behaviors can play a role in the long-term inflammatory response. Researchers have commonly used immunohistochemistry, western blotting and gene sequencing to establish the cellular processes behind UC relapse and remission. However, because these destructive methods necessitate the removal of a sample, they can only be used on non-living tissues. The use of minimally invasive approaches to evaluate the *in vivo*, longitudinal effects of UC on the mucosa in the colon is gaining popularity among clinicians and researchers. We have created a dextran sulfate sodium-induced model of UC in C57 mice based on the work of Wirtz et al., and a minimally invasive imaging modality to explore the changes in mucosal tissue during “active” and “in remission” UC. Briefly, C57 mice were given dextran sulfate sodium (DSS) dissolved in water in 5-day cycles with a remission/recovery period of 10 days. After 7 days post-DSS treatment and 7 days post-recovery, mice were anesthetized and exploratory endoscopies were performed to assess the mucosal changes that occur during the “active” and “remission” periods of UC. Value of protocol:

• Minimally invasive induction of ulcerative colitis in a murine mouse model.
• Minimally invasive longitudinal monitoring of “active” and “in remission” ulcerative colitis.
• Our endoscopic based imaging modality can be used to validate the induction of ulcerative colitis and the potential treatment response for pre-clinical trials.

## Graphical abstract

**Figure:**
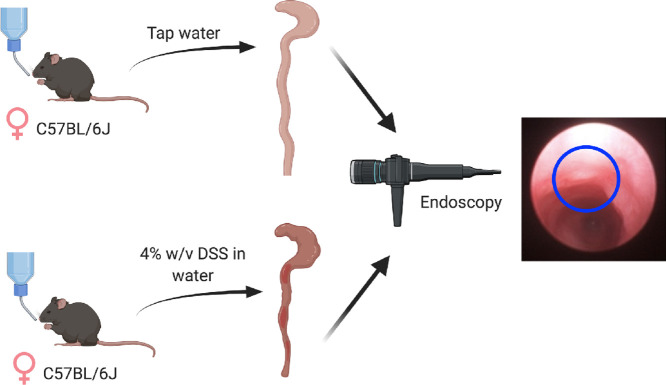

Specifications Table

**Table:** 

**Subject Area:**	Engineering
**More specific subject area:**	*Tissue biology; endoscopic imaging*
**Method name:**	*Dextran Sulfate Sodium-Induced Ulcerative Colitis*
**Name and reference of original method:**	*Chemically induced mouse models of intestinal inflammation* *Stefan Wirtz, Clemens Neufert, Benno Weigmann and Markus F. Neurath* Citation: Wirtz, S., Neufert, C., Weigmann, B. et al. Chemically induced mouse models of intestinal inflammation. Nat Protoc 2, 541–546 (2007). https://doi.org/10.1038/nprot.2007.41
**Resource availability:**	*N/A*

## Method details

*Safety Warning*: DSS induces epithelial cell breakdown when ingested. Follow Material Safety Data Sheet for correct handling and storage.

### Materials and reagents

A. *Reagents*
  1. Dextran Sulfate Sodium Salt Colitis Grade: MW 36,000–50,000 (MP Biomedicals, Catalog Number: 02,160,110-CF).
  2. 100 mL PBS tablets (VWR, Catalog Number: 97,062–730).
B. *Endoscopy Equipment*
  1. Karl Storz COLOView System.
  2. MVR Pro HD Recorder (Karl Storz, Catalog Number: MVR-409).
  3. Aquarium pump with ¼” plastic tubing
  4. Examination sheath (Karl Storz, Catalog Number: 61,029 D).
    i. This examination sheath is made for the diameter of a mouse colon. Karl Storz provides options for examination sheaths to accommodate for the diameter of a rat colon.
  5. Karl Storz Telescope (Karl Storz, Catalog Number; 64,301 AA).
  6. IMAGE1 S^TM^ H3-Z Three-Chip Full HD Camera Head (Karl Storz, Catalog Number: TH100).

### Procedures

#### DSS solution preparation and administration

1. During the acclimation period (usually 7 days) for the animals, measure how much water the mice drink in a day. This will depend on the mouse strain as well as the number of mice per cage.
2. Calculate the amount of solution required for the week of administration. DSS cycles can be 5 to 7 days.
  a. Use the w/v % of desired DSS concentration to calculate how much water and DSS is needed.
    i. For example, to make 4% DSS solution, add 1.2 g of DSS to 30 mL of tap water.
3. Mix DSS with the water inside a fume hood.
4. Divide all daily solutions into large conical tubes for each mouse cage.
5. Store all containers in a 4 °C fridge.
6. After the acclimatation period has passed, add the appropriate amount of DSS solution into each water bottle in each cage.
  a. Keep the DSS solution in the water bottle for three days. After three days, replace the DSS solution with fresh and change the solution every two days up until the DSS cycle is finished.

#### Procedure area setup

1. Attach the heating pad to the water heater. Turn the water heater on to 42°Celsius.
2. Connect the air pump.
3. When the heating pad is warm, wrap a large Kimwipe around the heating pad.

#### Endoscope setup (Figs. 1A and 2)

1. Obtain the Karl Storz telescope and insert it into the examination sheath.
2. Rotate the latch on the examination sheath to lock it into place.
3. Connect the camera into the Image 1 Hub and attach the end of the telescope to the camera.
4. Turn on the Image 1 Hub and verify you have an image on the computer monitor.
5. Connect the Optical Fiber into the Xenon 300 and at the side of the telescope.
6. Turn on the Xenon 300 and set the brightness to 75%.
7. Connect the air pump hose to the left insufflation port on the examination sheath.
8. Make sure the valves on the ports are turned on.
9. Turn on the MVR Pro HD recorder and start a New Procedure.

#### Endoscopy

1. Turn the valve on the oxygen tank and the vaporizer of the anesthesia system.
2. Place the mouse into an anesthesia chamber, make sure the hose valve connected to it is turned on, while the hose valve connected to the nose cone is off.
3. After the breathing pattern slows significantly, turn on the valve leading to the nose cone and move the mouse to the heating pad.
  a. **Note**: If the mouse is not fully anesthetized, place the mouse back in the anesthesia chamber. If the mouse were to wake up during the procedure, it is possible that the endoscopy can cause mucosal damage.
4. Place the mouse's nose in the cone.
5. Close the valve leading to an anesthesia Chamber.
6. Place GenTeal (severe) on the mouse's eyes.
7. Fill the 10 mL syringe with 3–5 mL of PBS to perform enemas. The PBS must be at room temperature. Enemas usually occur 5 min before the start of the endoscopy procedure. This step usually takes approximately 1–3 min.
  a. **Note**: If a mouse that has not had received DSS, an enema can be used to flush out fecal matter.
  b. **Note**: If the mouse has been on DSS, we do not recommend using the enema immediately.
8. Before entering with the telescope, place a few drops of moderate GenTeal gel on the protective insert that goes into the Examination Sheath.
9. Enter the colon with the endoscope.
  a. **Note**: The inflammation due by DSS can make it difficult to progress the endoscope into the colon. Insufflation while trying to enter may alleviate this problem.
    1. Alternatively, placing the tip of the scope just inside the anus and turning the scope to the side can also make entrance easier.
10. Place a finger on the insufflation port opposite the air hose to direct air into the colon. **Do not constantly keep your finger over the port as the mouse may retain the air for a while after it wakes up.**
  a. **Note**: As the operator progresses into the colon, inflammation and intestinal narrowing of the lumen can cause mucosal tearing without the proper insufflation. Mucosal tearing can lead to bleeding and potential death.
11. Progress as far as possible into the mouse's colon and then begin to retract.
12. As the telescope is retracting, ensure an assistant is taking pictures and videos of the whole retraction. Take note of the location of any ulcers.
  a. DSS ulcers tend to appear in the distal colon more frequently, so it is usually unnecessary to progress the scope all the way to the end of the large intestine.
13. In the event that the camera lens becomes dirty or fogged over, clean the lens by spraying the lens paper with 70% ethanol and wiping the lens.

## Expected results

### DSS-Induced ulcerative colitis

The scope of this article is on the administration of dextran sulfate sodium to induce ulcerative colitis and longitudinal monitoring of C57BL/6J mice. Briefly, C57BL/6J mice (Jackson Labs) were given 4% DSS (w/v) water solution for five days ad *libitum* and were given 10-days of recovery (Fig. 2). Weight loss was recorded daily as shown in our preliminary data (Fig. 3D). Through the course of a DSS “active” cycle, the average weight loss remained the same. Starting 24 h post-DSS administration, the average weight loss in the mice decreased significantly for two days before rebounding to approximately its original weight before starting the next “active” DSS cycle. Interestingly, approximately 40 days after the initial administration of DSS, the average weight loss for the DSS treated group showed a significant difference in weight versus that of their control counterparts. From days 40 to 76, the weight of the DSS group remained lower than that of the control group.

**Fig. 1:**
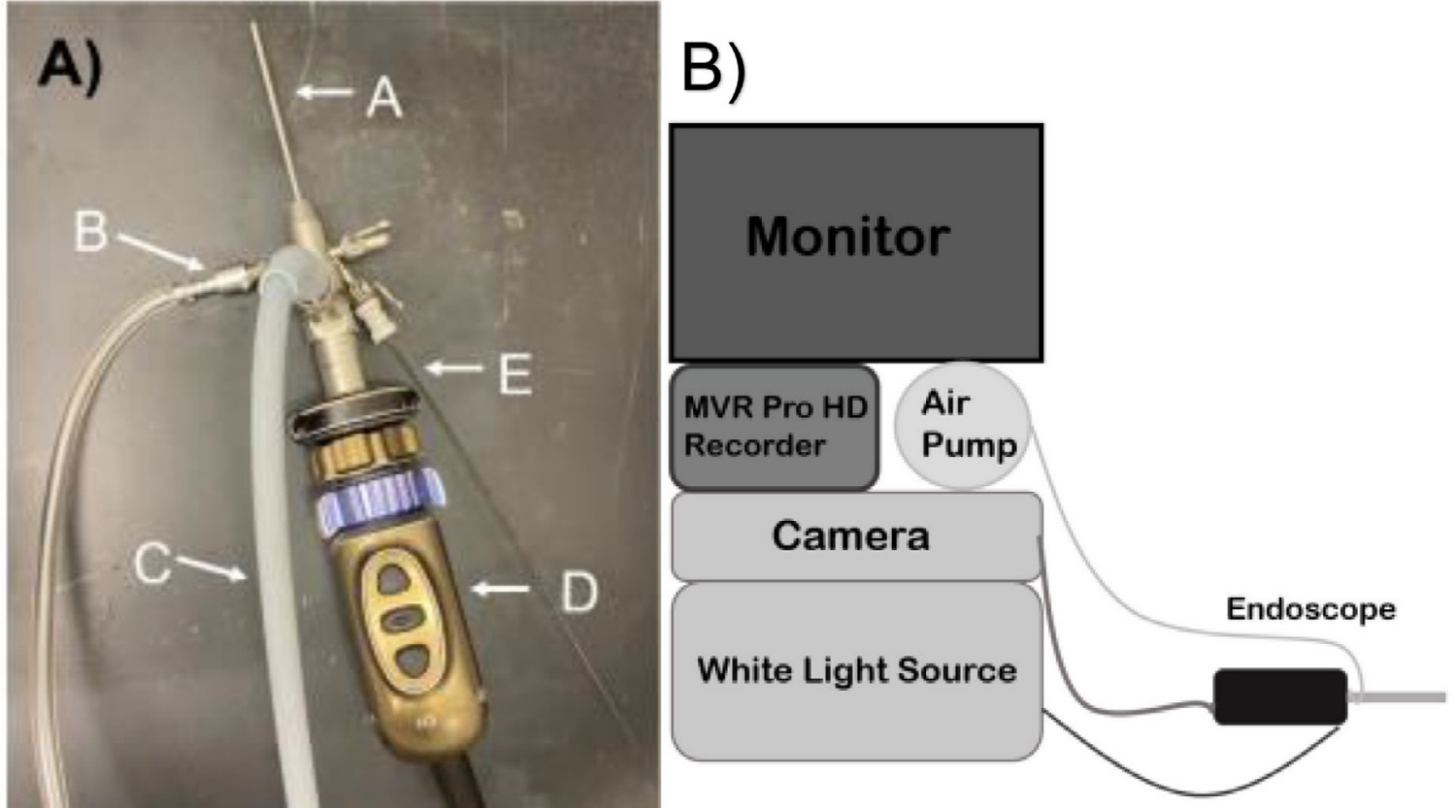
(A) Endoscope Setup: A – examination sheath B - Air Pump Hose C – Optical fiber D – Camera E – 1 mm DRS probe. (B) Diagram of Karl Storz COLOView system setup.

**Fig. 2:**
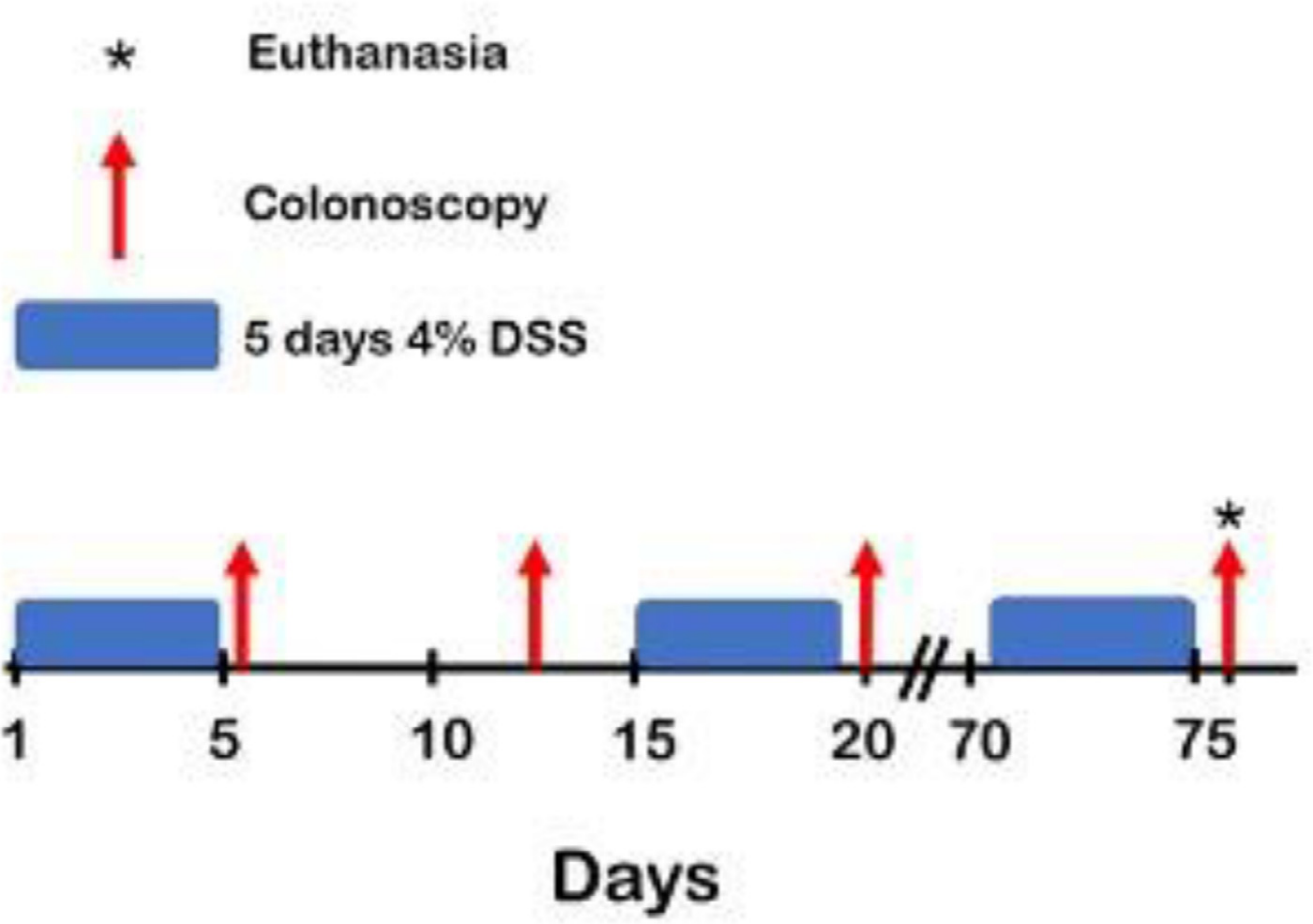
Sample Timeline of DSS administration with colonoscopies. Control mice are scoped every week without any DSS. DSS treated mice receive 5 days of 4% DSS with a 10-day recovery period between each cycle. This simulates the “active” and “remission” cycling of UC. DSS was administered for 12 weeks and the final colonoscopy and euthanasia occurred on day 76.

**Fig. 3:**
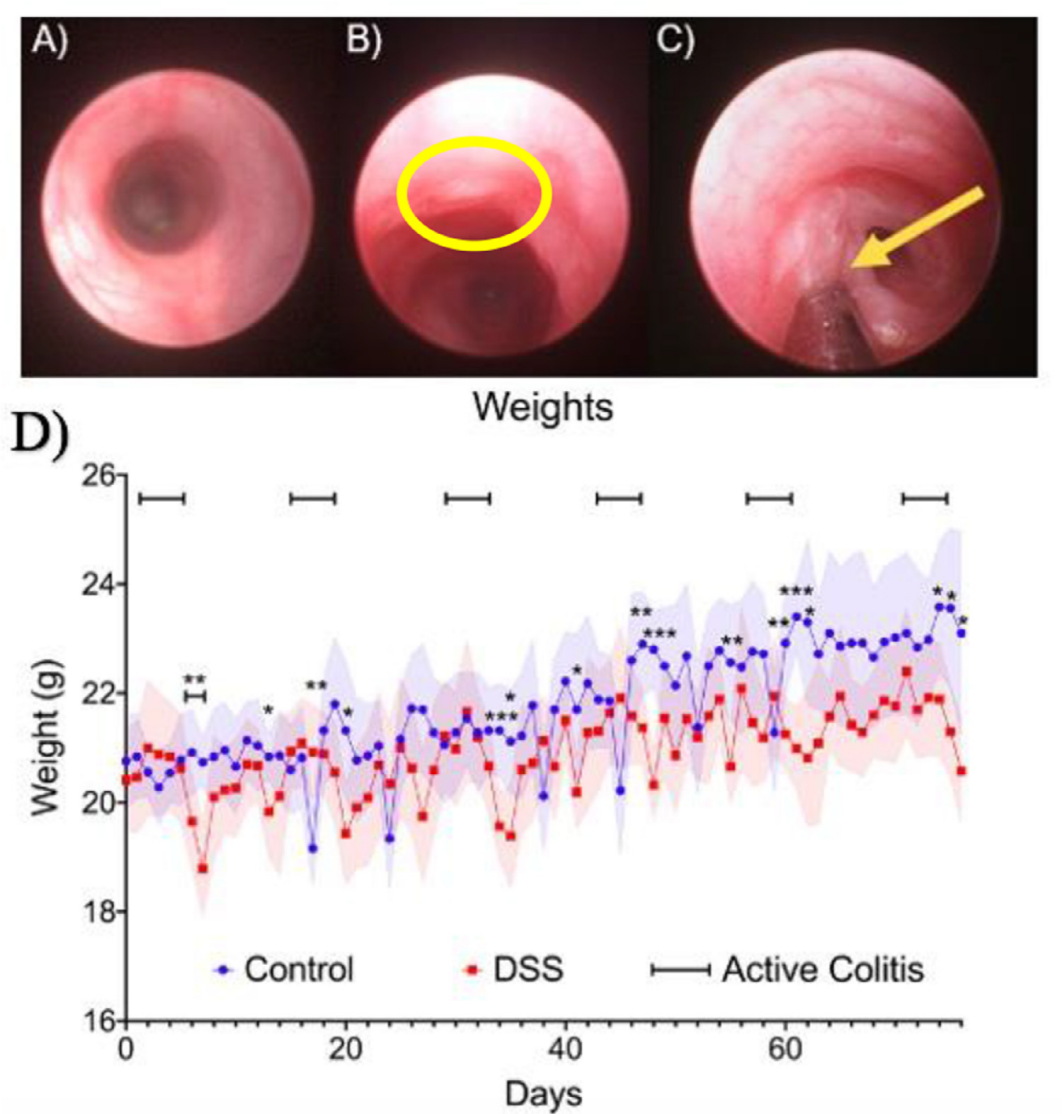
(A) Normal colon mucosa. (B) DSS-induced ulcer in the mucosa. (C) Optical probe making contact with an ulcer 76 days post-DSS treatment. (D) Longitudinal weight changes from C57BL/6J control (*n* = 5) and DSS induced mice (*n* = 9). Raw weight plots were made in GraphPad Prism©. (* *p* ≤ 0.05, ** *p* ≤ 0.01, *** *p* ≤ 0.0001, Mann-Whitney test).

### Endoscopic results

Developing ulcers were monitored during “active” and “remission” stages using a commercial colonoscope unit (Karl Storz COLOView) (Fig. 3). Representative images are shown in Fig. 3A–C. Fig. 3A is a representation of normal mucosa with no damage to the epithelial monolayer that lines the large intestine, which indicates the results seen after the first DSS cycle. In Fig. 3B, the highlighted region shows degradation of the mucosal lining. It is said that this is due to DSS carrying a “highly negative charge contributed by sulfate groups”, which can be toxic to the colonic epithelia. This induces the “erosion” of the epithelial barrier in the mucosa [1,2]. Fig. 3C shows an optical probe making contact with a developing ulcer. This probe was deployed through a biopsy port of the examination sheath of the COLOView system [3]. This allows for full visualization of the colon to determine the proper position to deploy the probe for data acquisition [3]. This optical probe, as described in Mundo et al. and in Greening et al., can be used to monitor hemodynamic tissue changes in regard to tissue oxygen saturation and total hemoglobin content [3], [4], [5]. Optical techniques such as diffuse reflectance spectroscopy can be used in conjunction with a colonoscope unit to help clinicians determine treatment response in diseases such as UC and colorectal cancer. To assess the amount of ulcerations, the Mayo Endoscopic Subscore can be used (Table 1). For the first half of the 12-week administration, the Mayo Subscore [6] for the C57 mice was between a 0 and 1. After approximately seven weeks, the Subscore increased from a 1 and averaged around a 2. This change in Mayo Subscore can be correlated with the weight loss shown after 50 days post-treatment in Fig. 3D. However, the Mayo Endoscopic Subscore is a qualitative measurement and doesn't give a clinician the hemodynamic changes that are occurring during the “active” and “remission” periods of UC. This indicates the need for additional optical tools, such as diffuse reflectance spectroscopy, that can monitor the changes in oxygen saturation and hemoglobin content within a developing ulcer and the changes to the surrounding tissue.

**Table 1:** Descriptions of the Mayo Endoscopic Subscoring system.

Mayo Endoscopic Subscore	Description
0	Normal mucosa or inactive disease
1	Mild activity (erythema, decreased vascular pattern, mild friability
2	Moderate activity (marked erythema, lack of vascular pattern, friability, erosions)
3	Severe activity (spontaneous bleeding, large ulcerations)

## Declaration of Competing Interest

The authors declare that they have no known competing financial interests or personal relationships that could have appeared to influence the work reported in this paper.
